# Risk of human papillomavirus-related cancers among kidney transplant recipients and patients receiving chronic dialysis - an observational cohort study

**DOI:** 10.1186/1471-2369-14-137

**Published:** 2013-07-08

**Authors:** Lars Skov Dalgaard, Ulrik Fassel, Lars Jørgen Østergaard, Bente Jespersen, Ole Schmeltz Søgaard, Søren Jensen-Fangel

**Affiliations:** 1 Department of Infectious Diseases, Aarhus University Hospital, Aarhus, Denmark; 2 Department of Nephrology, Aarhus University Hospital, Aarhus, Denmark

## Abstract

**Background:**

Individuals with end-stage renal disease (ESRD) have excess risk of various cancer types. However, the total burden of human papillomavirus-related cancers remains unknown.

**Methods:**

We performed a nationwide observational cohort study during 1994–2010.

For each person with ESRD, we sampled 19 population controls (without ESRD) matched on age, gender and municipality. Participants were followed until first diagnosis of human papillomavirus-related cancer, death, emigration, or 31 December 2010, whichever came first.

Human papillomavirus-related cancers were extracted from Danish medical administrative databases. We considered cancers of the cervix, vulva, vagina, penis, anus, and subsets of head and neck cancers as human papillomavirus-related. We calculated incidence rates of human papillomavirus-related cancer and used Poisson regression to identify risk factors for human papillomavirus-related cancer.

**Results:**

Among 12,293 persons with ESRD and 229,524 population controls we identified 62 and 798 human papillomavirus-related cancers, respectively. Incidence rates of human papillomavirus-related- cancer were 102 per 100,000 person-years (95% confidence interval [CI]; 79.5-131) among persons with ESRD and 40.8 per 100,000 person-years (95% CI; 38.1-43.7) among population controls. ESRD patients had 4.54 (95% CI, 2.48-8.31) fold increased risk of anal cancer and 5.81 fold (95% CI; 3.36-10.1) increased risk of vulvovaginal cancer. Adjusted for age, comorbidity, and sex, ESRD patients had 2.41 (95% CI; 1.83-3.16) fold increased risk of any human papillomavirus-related cancer compared with population controls. Compared with dialysis patients renal transplant recipients had an age-adjusted non-significant 1.53 (95% CI, 0.91-2.58) fold higher risk of human papillomavirus-related cancer.

**Conclusions:**

Persons with ESRD have excess risk of potentially vaccine-preventable human papillomavirus-related cancers.

## Background

Oncogenic types of human papillomavirus (HPV) can cause cancer of the cervix, vagina, vulva, penis, anus, and a subset of cancers of the head and neck [1-3]. Among persons infected with human immunodefiency virus (HIV), the immunosuppression caused by HIV contributes to the well described excess risk of HPV-related cancer [4,5]. In patients with end-stage renal disease (ESRD) immunosuppressive therapy is used to prevent transplant rejection among transplant recipients and occasionally for treatment of the underlying cause of renal failure among patients on dialysis. Nutritional deficits and uraemia in itself also contributes to the complex dysfunction of both the innate and adaptive immune system that characterizes ESRD patients on dialysis [6-8]. Among transplant recipients, previous studies have shown a markedly increased risk of a wide range of cancers including infection-related cancers [9-13]. Among ESRD patients receiving dialysis, the excess cancer risk appears to be less pronounced and primarily correlated to cancers with a known or suspected viral aetiology [10,14,15]. The immune dysfunction in ESRD patients may explain the increased risk of infection-related cancers [10,14].

While most studies have focused on heterogeneous cancer types among patients with ESRD, few studies have assessed the total burden of HPV-related cancers. In light of the recent development in prophylactic HPV vaccination, the interest in HPV-related cancers has been renewed. Vaccines against the two most important oncogenic types of HPV (16 and 18) are now licensed. Among younger healthy individuals HPV vaccination lowers the risk of pre-cancer lesions of the cervix and the risk of anal, vulva, vaginal, and penile infections, with the HPV-types included in the vaccines [16-20]. Further, research in novel vaccines for treatment of existing lesions caused by HPV has shown promising results [21].

A recently published large American cohort study found no increased risk of cervical cancer among solid organ transplant recipients, which contrasts the findings reported by Vajdic and colleagues among Australian kidney transplant recipients [9,10]. These findings may be explained by differences in screening procedures prior to transplantation and by differences in study populations. In several studies, HPV-related cancer diagnoses have been grouped with non-HPV-related cancer diagnoses [10-14,22].

The benefit and cost-effectiveness of HPV immunization will depend on the total burden of HPV-related cancer, the proportion of ESRD patients with active HPV infection at the time of vaccination, and the immunogenicity of the vaccines among these patients. Thus, improved knowledge of the epidemiology of HPV-related cancers among ESRD patients are of great importance for the decisions of “if” “who”, and “when”, to vaccinate.

The main objectives of this Danish nationwide population-based cohort study were to compare the risk of HPV-related cancers in the general population with that of patients with ESRD, and to identify risk factors for HPV-related cancers in patients with ESRD.

## Methods

### Study design and setting

We performed a population-based, observational cohort study among ESRD patients in Denmark during 1 January 1994–31 December 2010. In Denmark, the prevalence of chronic renal failure requiring renal replacement therapy, i.e. dialysis or transplantation is 0.08% and in 2010 approximately 4,700 persons were living with ESRD [23]. Treatment of ESRD is restricted to 15 hospital departments, 4 of which had status as transplantation centers during the study. In Denmark, the tax-funded health care system provides free of charge treatment for all patients with ESRD. Since 1990, all departments treating ESRD patients report data to the Danish Nephrology Registry (DNR). The DNR contains information on e.g. cause of renal disease, date of first replacement therapy for ESRD, treatment modality, and treatment complications. The DNR has a completeness of 97.2% [24].

### Participants

We defined ESRD patients as patients treated with renal replacement therapy (dialysis or transplantation) for at least 90 days. Prevalent ESRD patients as of 1 January 1994 or incident thereafter, who were resident in Denmark and at least 14 years of age at the time of their first treatment for ESRD were identified in the DNR and included in the study. For each ESRD patient we sampled 19 population controls matched on age (within 1 year of date of birth), municipality, and gender from the Civil Registration System (CRS) at the day of inclusion in the ESRD registry. Since 1968, the CRS has provided all Danish citizens with a unique 10 digit civil registration number, which allows for accurate linkage between different databases [25].

We obtained information on vital status, sex, date of birth, date of death, and residence of ESRD patients and their population controls from the CRS, which is updated within a week of person’s birth, death, or emigration. Population controls were used for comparative analysis. ESRD patients and population controls diagnosed with HPV-related cancer prior to study participation were excluded.

### Variables and data sources

#### Definition of HPV-related cancer

We classified cancers of the anus, vulva, vagina, cervix, penis, and a subset of head and neck cancers as HPV-related [2]. Cancer diagnoses were coded according to the 10^th^ edition of the International Classification of Diseases (ICD-10) as outlined in Table 1. For head and neck cancers, we used the classification published by Ryerson et al. in order to restrict our analyses to the cancer sites most strongly associated with HPV [26].

**Table 1:** **Diagnoses in the 10**^**th **^**edition of the International Classification of Diseases (ICD-10) considered human papillomavirus-related**

**Anatomical region**	**ICD-10 diagnoses considered related to human papillomavirus**
Head and neck^a^	C01.9, C02.4, C02.8, C09.0, C09.1, C09.8, C09.9, C10.2, C10.8, C10.9, C14.0, C14.2, C14.8
Cervix	C53.0- C53.9
Vulva and vagina	C51.0- C52.9
Penis	C60.0-C60.9
Anus	C21.0 -C21.8

^a^based on classification used by Ryerson et al.[26].

#### Identification of patients with HPV-related cancer

The Danish National Registry of Patients (DNRP) contains information of all hospital admissions in Denmark since 1977 and all outpatient contacts since 1995 [27,28]. Variables include civil registration number, dates of admission and discharge, and discharge diagnoses. Since 1994, diagnoses have been coded according to the ICD-10. During 1977–1993 the 8^th^ version (ICD-8) was used. The Danish Cancer Registry (DCR) contains data on incident cancers in Denmark since 1943.The DCR is nationwide and contains ICD-10 diagnoses (as part of the ICD-0 classification) since 1978 [29]. Original diagnoses from 1978–1993 have been electronically recoded from ICD-7 to ICD-10. We classified the earliest date of a HPV-related cancer diagnosis as the date of the first diagnosis of a HPV-related cancer. We used both the DNRP and the DCR to identify HPV-related cancer. In a random sample of 100 persons with a HPV-related cancer, 75% were identified in the DCR while 25% was identified in the DNRP alone.

#### Age, sex, cause of renal failure and comorbidity

Based on date of birth in the CRS we generated a time varying covariate *age (*14*–*49, 50–64, and ≥65 years). Information on cause of renal failure was obtained from the DNR database. Causes of renal failure were categorized as follows: *Glomerulonephritis*, *Diabetes Mellitus types I and II*, *Chronic Interstitial Nephritis, Hypertension and Chronic renal failure without specification, Polycystic Kidney Disease*, *Vasculitis*, and *Other.* The Charlson’s Comorbidity index (CCI) was used to quantify comorbidity. The CCI is a weighted index based on 19 major disease categories each assigned with a weight (1, 2, 3, or 6). CCI is a validated measure of co-morbidity [30,31]. We did not include renal diagnoses in the CCI score in this study. Based on discharge diagnoses obtained from the Danish National Registry of Patients (DNRP) we calculated CCI for each ESRD patient and his/her population controls at the time of study entry. We used CCI at the time of first treatment for ESRD to create 3 levels of co-morbidity: low (CCI = 0) medium (CCI = 1-2) and high (CCI ≥3).

### Statistical analysis

#### Time at risk

For each ESRD patient and his/her population controls time at risk started at 1 January 1994 or the date of first treatment for ESRD, whichever came last. Time at risk ended at the date of first diagnosis of a HPV-related cancer, death, emigration, or 31 December 2010, whichever came first.

#### Incidence rates

We calculated incidence rates (IRs) and 95% confidence intervals (95% CIs) of HPV-related cancers with and without stratification for the five anatomic regions outlined in Table 1. We calculated incidence rates of HPV-related cancer with and without prevalent ESRD patients and only minor differences in risk estimates were found. We therefore chose to include both prevalent and incident ESRD patients in our final analysis. Temporal changes in IRs of first episodes of HPV-related cancers were analysed in following intervals 1994–97, 1998–01, 2002–2005, and 2006–2010. Incidence rate ratios (IRRs) with corresponding 95% CIs were calculated for comparison between ESRD patients and population controls. In a separate analysis IRs of HPV-related cancer were compared among two subgroups of ESRD patients: 1) transplant recipients with functioning grafts and 2) dialysis patients*.* In these analyses, renal replacement therapy i.e. dialysis and transplant recipients with functioning grafts was treated as a time varying covariate. Thus, if the mode of renal replacement therapy was changed during follow-up, a person would contribute to risk time in both subgroups. IR’s were compared between the two subgroups with and without stratification for age. For the latter analyses Poisson regression was used: Adjustment for sex, comorbidity and cause of renal failure only caused minor effect on the risk estimates and therefore neither of these variables were included in the final analyses.

#### Risk factor analysis

We used Poisson regression to identify potential risk factors for HPV-related cancer among ESRD patients. The following variables were included: Sex, age (14–49, 50–64, and ≥65 years), and the CCI index score. Among the study population 24 patients with ERSD and 227 population controls were HIV positive. Among HIV-positive persons in our study population, only two (population controls) developed a HPV-related cancer during the study period and we therefore chose not to adjust for HIV status in our analyses. While age and sex are well-known risk factors for cancer, the CCI is not an established risk factor for HPV-related cancer. We therefore calculated adjusted risk estimates with and without the CCI. In the risk factor analysis, we did not stratify for anatomical location of HPV-related cancers.

We used the Stata® statistical software (Statacorp, College Station, Texas) version 11.0 for statistical analysis. The study was approved by the Danish Data Protection Agency.

## Results

### Participants

We excluded 92 ESRD patients (and their matched controls) diagnosed with a HPV-related cancer prior to study participation. A total of 442 population controls diagnosed with HPV-related cancer prior to study participation were also excluded. Persons who died or emigrated before the study period did not contribute to risk time. Consequently 12,293 ESRD patients and 229,524 population controls were included in the study.

### Descriptive data

Table 2 displays basic characteristics of the study population. Males comprised 62.5% of the ESRD population. The 12,293 ESRD patients and 229,524 population controls provided 60,813 and 1,955,900 person-years of risk time respectively.

**Table 2:** Baseline characteristics of the study population

	**End-stage renal disease**	**Population Controls**	**P**
Number of participants	12,293	229,524	-
Male sex, n (%)	7,683 (62.5)	143,622 (62.6)	0.9
Age at indexdate^a^, n (%)			
14-49	3,516 (28.6)	64,464 (28.1)	0.5
50-64	3,630 (29.5)	68,413 (29.8)	
≥65	5,147 (41.9)	96,647 (42.1)	
Comorbidity level at indexdate^b^			
Low	4,231 (34.4)	169,330 (73.8)	<0.001
Medium	3,663 (29.8)	46,539 (20.3)	
High	4,399 (35.8)	13,655 (6.0)	
Cause of end-stage renal disease			
Glomerulonephritis	1,751 (14.2)	-	NA
Diabetes I + II	2,595 (21.1)	-	
Chronic interstitial nephritis	1,496 (12.2)	-	
Hypertension and Chronic renal			
failure without specification	4,019 (32.7)	-	
Adult polycystic kidney disease	969 (7.9)	-	
Vasculitis	438 (3.6)	-	
Other	1,025 (8.3)	-	
Transplant history			
No transplantation	8,978 (73.0)	-	NA
≥1 Transplantation	3,315 (27.0)	-	

^a^Indexdate: Date of first treatment for end-stage renal disease and date of sampling for controls.

^b^Three levels of comorbidity was created based on Charlson comorbidity index score (CCI) at the date of first treatment with renal replacement therapy “Low” (CCI = 0), “Medium” CCI 1–2, and “High” CCI > 2. Renal diagnosis was not included in the CCI score in this study.

Median time at risk was 3.35 (maximum 17.0) years and 7.85 (maximum 17.0) years among ESRD patients and population controls, respectively. Median age of ESRD patients at the date of their first treatment with renal replacement therapy was 61.6 years.

### Outcome data

During the study period we identified a total of 62 cases of HPV-related cancers among ESRD patients and 798 cases among population controls (Table 3).

**Table 3:** Crude incidence rates of first episodes of Human papillomavirus-related cancers among persons with end-stage renal disease and population controls during 1994-2010

**Anatomical region**	**Group**	**No.**	**Risk time**^**a**^	**Crude IR**^**b **^**(95% CI)**	**IRR (95% CI)**	**P value**
Head and neck^c^	ESRD	18	0.608	29.6 (18.6-47.0)	1.77 (1.10-2.84)	0.02
	Controls	328	19.559	16.8 (15.1-18.7)		
Cervical^d^	ESRD	12	0.229	52.3 (29.7-92.1)	1.81 (1.01-3.23)	0.05
	Controls	221	7.644	28.9 (25.3-33.0)		
Vulva and vagina^d^	ESRD	15	0.229	65.4 (39.4-108)	5.81 (3.36-10.1)	<0.001
	Controls	86	7.644	11.3 (9.11-13.9)		
Penis^e^	ESRD	5	0.379	13.2 (5.49-31.7)	2.02 (0.82-4.98)	0.1
	Controls	78	11.915	6.55 (5.24-8.17)		
Anal	ESRD	12	0.608	19.7 (11.2-34.7)	4.54 (2.48-8.31)	<0.001
	Controls	85	19.559	4.35 (3.51-5.38)		
All HPV-related	ESRD	62	0.608	102 (79.5-131)	2.50 (1.93-3.24)	<0.001
	Controls	798	19.559	40.8 (38.1-43.7)		

Abbreviations: *HPV* Human papilloma virus, *No.* number of HPV-related cancers, *CI* confidence interval, *IR* incidence rate, *IRR* incidence rate ratio, *ESRD* end-stage renal disease.

^a^ 100,000 years.

^b^ Per 100,000 person-years.

^c^ HPV-related head and neck cancer sites as outlined in Table 1.

^d^ Only females contributed to risk time.

^e^ Only men contributed to risk time.

### Main results

**Figure 1:**
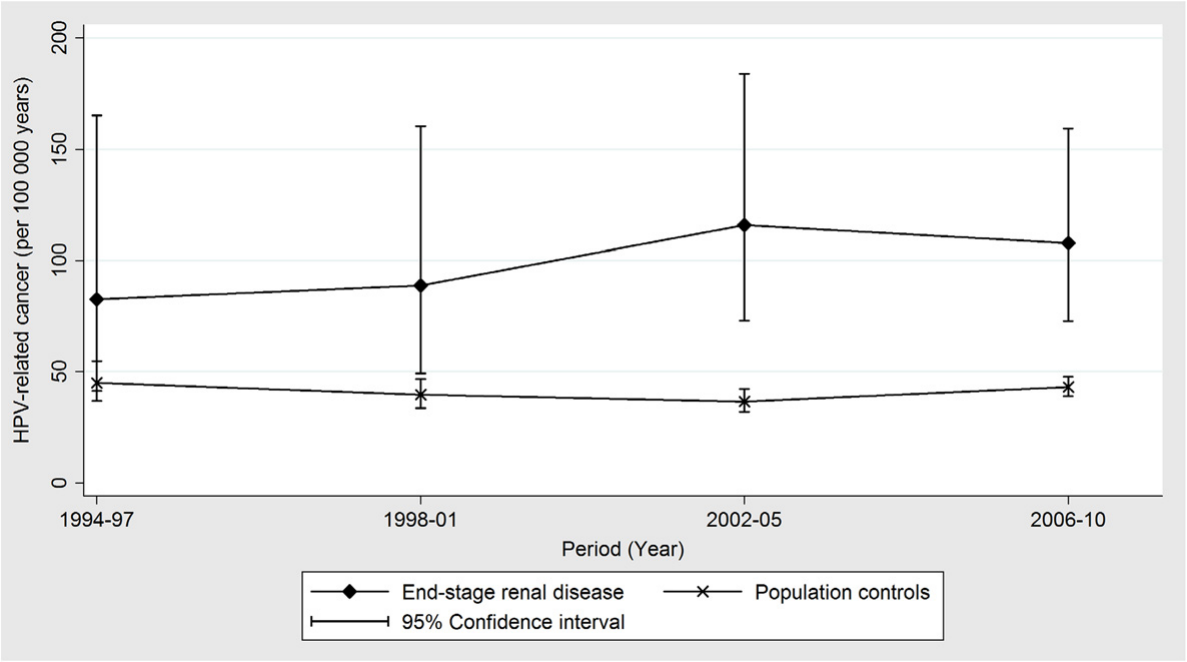
Temporal trends in crude incidence rates of human papillomavirus-related cancers among patients with end-stage renal disease and a matched background population.

Table 3 outlines crude IRs of first diagnosis of HPV-related cancer among ESRD patients and population controls. The overall IRs of first diagnosis of a HPV-related cancer was 102 (95% CI, 79.5-131) per 100,000 person-years and 40.8 (95% CI, 38.1-43.7) per 100,000 person-years among ERSD patients and population controls, respectively. The IR of all HPV-related cancers among ESRD patients was 2.50 (95 CI%, 1.93-3.24) fold higher than among population controls. The IRs of first episode of HPV-related cancer were significantly higher among ESRD patients compared to the control population in 4 of 5 anatomical regions, most markedly for anal cancer (IRR 4.54; 95% CI, 2.48-8.31) and vulva/vaginal cancer (IRR 5.81; 95% CI, 3.36-10.1). The IRRs of cervical cancer and cancers at HPV-related head & neck sites were 1.81 (95% CI, 1.01-3.23) and 1.77 (95% CI, 1.10-2.84) respectively. Figure 1 shows a modest non-significant increase in IRs of first diagnosis of HPV-related cancers among ESRD patients over the study period (p = 0.57), whereas IRs among population controls remained stable.

Among ESRD patients, transplant recipients with functioning grafts had an unadjusted 1.83 (95% CI, 1.11-3.01) fold higher risk of HPV-related cancer compared to dialysis patients. When adjusted for age IRR fell to 1.53 (95% CI, 0.91-2.58) comparing transplant recipients to dialysis patients (Table 4).

**Table 4:** Risk of human papillomavirus-related cancer among patients with end-stage renal disease by type of renal replacement therapy

	**Dialysis**		**Graft function**			
	**No.**	**IR (95% CI)**^**a**^	**No**	**IR (95%CI)**^**a**^	**IRR (95% CI)**	**P value**
Head and neck^b^	8	21.4 (10.7-42.7)	10	42.9 (23.1-79.6)	2.00 (0.71-5.85)	0.1
Vulva and vagina	5	35.0 (14.5-84.0)	10	116 (62.3-215)	3.31 (1.13-9.69)	0.03
Cervix	8	55.9 (28.0-112)	4	46.3 (17.4-123)	0.83 (0.25-2.75)	0.8
Anal	5	13.3 (5.55-32.1)	7	30.0 (14.3-62.9)	2.25 (0.71-7.08)	0.2
Penis	3	13.0 (4.18-40.2)	2	13.6 (3.40-54.4)	1.05 (0.18-6.29)	0.9
All HPV-related	29	77.4 (53.8-111)	33	141 (100–198)	1.83 (1.08-3.12)	0.02

Abbreviations: *No.* number cancers, *CI* confidence interval, *IR* incidence rate, *IRR* unadjusted incidence rate ratio, *IR* incidence rate.

^a^ Per 100,000 person-years.

^b^ HPV-related head and neck cancer subsites as outlined in Table 1.

### Risk factors for HPV-related cancer

Table 5 illustrates potential risk factors for HPV-related cancers among ESRD patients and population controls. Among ESRD patients female sex (IRR 2.11; 95% CI, 1.27-3.48) and age 50–64 years (IRR 2.39; 95% CI, 1.30-4.39) were risk factors for HPV-related cancer. Among population controls, female sex (IRR 1.54; 95% CI, 1.34-1.77), age (50–64 years: IRR 2.14; 95% CI, 1.67-2.75 and ≥65 years: IRR 2.75; 95% CI, 2.18-3.48) and co-morbidity level (medium: IRR 1.41; 95% CI 1.18-1.69 and high: IRR 2.49 (95% CI; 1.89-3.28) were all risk factors for HPV related cancer. Interestingly, the IR of HPV–related cancer among ESRD patients was markedly higher at age 50–64 years compared to age 14–49 years and age ≥65 years. When adjusted only for age and sex we found an IRR of 2.76 (95% CI, 2.13-3.58) of HPV-related cancer compared to population controls.

**Table 5:** Incidence rates of first episodes of human papillomavirus-related cancers by potential risk factors among persons with end stage renal disease and population controls during 1994-2010

**Variable**	**No.**	**IR**^**a **^**(95% CI)**	**Unadjusted IRR (95% CI)**	**Adjusted IRR**^**b **^**(95% CI)**^**b**^	**P**^**c**^
Total					
Controls	798	40.8 (38.1-43.7)	1(reference)	1 (reference)	-
ESRD	62	102 (79.5-131)	2.50 (1.93-3.24)	2.41 (1.83-3.16)	<0.001
Sex Controls					
Men	404	33.9 (30.8-37.4)	1 (reference)	1 (reference)	-
Women	394	51.5 (46.7-56.9)	1.52 (1.32-1.75)	1.54 (1.34-1.77)	<0.001
ESRD					
Men	27	71.3 (48.9-104)	1	1	-
Women	35	153 (110–213)	2.14 (1.30-3.54)	2.11 (1.27-3.48)	0.004
Age (years) Controls					
14-49	83	18.8 (15.2-23.3)	1 (reference)	1 (reference)	-
50-64	242	40.2 (35.5-45.7)	2.14 (1.67-2.75)	2.11 (1.64-2.71)	<0.001
≥ 65	473	51.8 (47.3-56.7)	2.75 (2.18-3.48)	2.51 (1.98-3.18)	<0.001
ESRD					
14-49	15	73.0 (44.0-121)	1 (reference)	1 (reference)	-
50-64	34	165 (118–231)	2.26 (1.23-4.15)	2.39 (1.30-4.39)	0.005
≥ 65	13	66.0 (38.3-114)	0.90 (0.43-1.90)	1.03 (0.48-2.19)	0.9
Comorbidity level ^d^ Controls					
Low	591	36.8 (34.0-39.9)	1 (reference)	1 (reference)	-
Medium	152	52.1 (44.4-61.1)	1.41 (1.18-1.69)	1.23 (1.02-1.47)	0.03
High	55	91.7 (70.4-119)	2.49 (1.89-3.28)	2.12 (1.60-2.81)	<0.001
ESRD					
Low	40	130 (95.1-177)	1 (reference)	1 (reference)	-
Medium	15	98.4 (59.3-163)	0.76 (0.42-1.37)	0.77 (0.42-1.41)	0.4
High	7	47.5 (22.6-99.6)	0.37 (0.16-0.82)	0.39 (0.17-0.87)	0.02

Abbreviations: *No* number of human papillomavirus-related cancers, *IR* incidence rate, *IRR* incidence rate ratio.

^a^ Per 100,000 person-years.

^b^ Adjusted for all variables in the table.

^c^ Calculated for the corresponding adjusted incidence rate-ratios.

^d^ 3 levels of comorbidity was created based on Charlson comorbidity index (CCI) score at the date of first treatment with renal replacement therapy “Low” (CCI = 0), “Medium” CCI 1–2, and “High” CCI > 2. Renal diagnosis was not included in the CCI score in this study.

## Discussion

In this nationwide, population-based study, we found that the risk of HPV-related cancer overall was 2.5 fold increased among ERSD patients compared to matched population controls. Interestingly, ESRD patients had a more than fourfold increased risk of anal cancer and female ESRD patients had a nearly six fold increased risk of cancers of vulva and vagina. Major risk factors for HPV-related cancer were age and gender. When adjusted for age, transplant recipients (with a functioning graft) had a modest 1.53 fold increased risk of HPV-related cancer compared to dialysis patients. Although not significant we cannot rule out an increased risk of HPV-related cancer among transplant recipients due to the low number of cancers in the two groups.

Our study is one of few population-based studies to investigate the burden of HPV-related cancer among unselected individuals with ESRD. The major strengths of our study are the use of population-based nationwide cohorts with high degrees of completeness and minimal loss to follow-up. Because we considered only the first diagnosis of HPV-related cancer our estimates were not biased by multiple episodes of HPV-related cancer occurring in highly susceptible individuals. Further, HPV-related cancer may increase the risk of development of ESRD and therefore lead to increased risk of HPV-related cancer among ESRD patients.

Our study had some limitations. The relatively low number of HPV-related cancer limited our opportunities to adjust for potential risk factors and the width of confidence interval complicates the comparison between transplant recipients and patients on dialysis. However, large follow-up time and careful matching yielded estimates of high statistical precision and validity.

We did not have access to information on use of immunosuppressive drugs among ESRD patients and therefore we could not assess the effect of individual drug regimens on the risk of HPV-related cancer. Likewise, we were unable to identify dialysis patients receiving immunosuppressive therapy for their renal disease. Whereas HPV is considered mandatory for development of cervical cancer the etiological fraction of HPV differ among other cancer types considered as HPV-related. If the etiological fractions of HPV are higher among ESRD patients than among population controls this may have led to underestimation of the importance of HPV among ESRD patients in our study. We restricted our analysis to the cancers most strongly associated with HPV in order to minimize the risk of misclassification of non-HPV-cancers as HPV-related. Whereas diagnoses in hospital discharge registries may be revised and therefore not reflect true cancers, cancer registries have a degree of underreporting. We chose to include cancers identified solely in the DNRP in this study. In the DCR, ICD-7 diagnoses were used until 2004. Subsequently diagnoses have been electronically recoded from ICD-7 to ICD-10. In terms of HPV-related head and neck sites, this may cause some misclassification during the recoding process due to the very specific cancer sites (ICD-10) used to identify these cancers. In these cases DNRP may be more accurate than the DCR. In the remaining cases the validity of cancers identified in the DNPR may be lower than cancers identified in the DCR. Previous studies have indicated high completeness of the DCR and DNP for breast cancer [32], urological cancers [33], colorectal cancers [34], and haematological cancers [35]. To ensure inclusion of all HPV-related cancers we therefore used both the DCR and DNPR.

In a previous study, Birkeland and colleagues reported a 8.6 fold increased risk of cervical cancer among female kidney transplant recipients [13]. In a more recent study, Vajdic and colleagues reported a two and a half fold increased risk of cervical cancer among both transplant recipients and patients on dialysis for ESRD. Shebl et al. reported a 2.12 fold increased risk of cervical cancer among elderly ESRD patients on dialysis [15]. We found a 1.78 fold increased risk of cervical cancer among female ESRD patients and incidence rates of cervical cancer were similar among dialysis patients and patients with functioning grafts. In Denmark, nationwide systematic screening for cervical cancer and pre-cancer lesions of the cervix was implemented during the early 1990’s [36,37]. Our results therefore reflect the risk of cervical cancer in a setting with systematic cervical screening offered free of charge for the study population. A recent American study by Engels and colleagues reported no increased risk of cervical cancer among solid organ transplant recipients [9].

For non-cervical HPV-related cancer among kidney transplant recipients standardized incidence rate-ratios reported by Engels were in concordance with our findings in ESRD patients and the findings by Vajdic and colleagues [9,10].

The cancer risk among dialysis patients is less well studied than among kidney transplant recipients. A previous study suggested that dialysis patients had a modest 1.18 fold increased cancer risk overall, whereas the risk of cervical cancer, penile cancer, and cancer of the oral cavity, was between 1.7 and 4.0 fold increased [14]. However, incomplete data on cancer diagnoses and significant variation in transplantation rates and renal diagnoses may have affected these results [14,38]. In a more recent study, Van Leeuwen et al. reported a rapid decline in risk of non-Hodgkin’s lymphoma, melanoma, and cancer of the lip, among kidney transplant recipients following transplant failure and reinstitution of dialysis [39]. Unfortunately, the study had only 7,104 person-years of follow-up during dialysis following transplant failure and only one case of HPV-related cancer was identified among graft-loss patients with consequent uncertainty in risk estimates for HPV-related cancer. Our results suggest that dialysis patients are at increased risk of HPV-related cancer which is in concordance with findings by Vajdic and colleagues [10].

Overall we estimate the external validity of our results to be high due to the population based design, choice of study period, the high quality of the databases used for the study, and the overall concordance with existing literature. Nevertheless differences in renal populations exist and should always be taken into account when applying our results to other renal populations.

Our results suggest that efforts to prevent HPV-related cancer should focus on dialysis patients as well as transplant recipients. Despite nationwide free of charge screening programs for cervical cancer, ESRD patients are still at increased risk of these cancers. Whether attendance to cervical screening differs among ESRD patients and the control population in our study population is unknown. Given the high IRs of vulvovaginal cancer it is important that the clinicians performing cervical smears are also observant to early signs of these cancers in ESRD patients For non-cervical HPV-related cancers no validated screening programs are currently available. Anal cancer screening have been considered among HIV infected men, but low sensitivity of cytology, high prevalence of anal intraepithelial neoplasia, and uncertainty of the natural history of these lesions complicates development of a screening program. For these cancers, early detection therefore relies on clinician’s and patients’ awareness on early signs and symptoms of cancer.

Two HPV vaccines are commercially available [40,41]. Both vaccines cover the most important oncogenic HPV-serotypes16 and 18, which are considered responsible for 70% of cervical cancers and the majority of non-cervical cancers caused by HPV [2]. In healthy individuals HPV-vaccination can prevent reactivation/reinfection with vaccine HPV serotypes to which they were seropositive and DNA negative at study enrolment [42,43]. The vaccines have no therapeutic effect against existing HPV-related lesions [17,44,45]. Neither of the vaccines has been formally tested in persons with ESRD but studies among HIV infected men and children have reported high rates of seroconversion. Our study illustrates that the total burden of HPV-related cancers is low among ESRD patients. Further the proportion of cancers caused by HPV infection prevalent at the time of diagnosis of ESRD patients is to our knowledge unknown. If this proportion is high the benefit of HPV vaccination is likely to be limited.

## Conclusions

Our study demonstrates that persons with ESRD have a markedly increased risk of HPV-related cancer compared to matched population controls. Patients with end-stage renal disease had a 4.5 fold increased risk of anal cancer and an almost 6 fold increased risk of vulvovaginal cancer. Transplant recipients only had a modest non-significant 1.53 fold increased risk of HPV-related cancer compared to dialysis patients. Age and female sex were most important risk factors for HPV-related cancer among persons with end-stage renal disease. Future studies of safety, immunogenicity, and efficacy of prophylactic HPV-vaccines among ESRD patients are needed to clarify the benefit of HPV-vaccination in this population.

## Competing interests

The authors declare that they have no competing interests.

## Authors’ contributions

SJF, BJ, and OSS conceived the study idea. SJF, OSS, LSD, BJ, LJØ and UF designed the study. OSS and BJ collected the data. LSD, OSS, and UFF analysed the data. All authors interpreted the data. LSD wrote the first draft, and all authors critically reviewed and edited the manuscript and approved the final version.

## Pre-publication history

The pre-publication history for this paper can be accessed here:

http://www.biomedcentral.com/1471-2369/14/137/prepub
